# Prevalence and associated factors of intestinal parasitic infections among asymptomatic food handlers working at Haramaya University cafeterias, eastern Ethiopia

**DOI:** 10.1186/s40557-018-0263-7

**Published:** 2018-08-22

**Authors:** Dadi Marami, Konjit Hailu, Moti Tolera

**Affiliations:** 1 Department of Medical Laboratory Sciences, College of Health and Medical Sciences, Haramaya University, P.O. Box 235, Harar, Ethiopia; 2 Higher Health Center, Haramaya University, P.O. Box 138, Dire Dawa, Ethiopia; 3 School of Public Health, College of Health and Medical Sciences, Haramaya University, P.O. Box 235, Harar, Ethiopia

**Keywords:** Intestinal protozoa, Helminth, Food handler, Sanitation, University, Cafeteria

## Abstract

**Background:**

Intestinal parasitic infections are major public health problems worldwide, with high prevalence in low income countries where substandard food hygiene practices are common. Asymptomatic food handlers with poor personal hygiene could be potential sources of parasitic infections. This study was aimed to assess the prevalence of intestinal parasitic infections and associated factors among asymptomatic food handlers working at Haramaya University cafeterias, eastern Ethiopia.

**Methods:**

A cross-sectional study was conducted among asymptomatic food handlers working at Haramaya University cafeterias from August 2015 to January 2016. Population proportion to size allocation and systematic random sampling techniques were used to identify the study participants. Stool samples were collected and examined simultaneouly using direct and modified formol ether concentration wet smear techniques. Data were entered and analyzed using SPSS version 20.0 software. Logistic regressions were applied to assess association between independent variable and intestinal parasitic infections. Statistical significance was declared at a *p*-value less than 0.05.

**Results:**

A total of 417 asymptomatic food handlers were enrolled in this study. Of these, females comprised 79.4%. Large proportion (39.3%) of food handlers were in the age group of 31–40 years. The overall prevalence of intestinal parasitic infections was 25.2% (95% CI: 18.3, 29.6). *Entamoeba histolytica/ dispar* (46.7%) and *A. lumbricoides* (14.3%) were the most frequent isolates. Having no formal education [AOR: 2.13, 95% CI: 1.24, 3.67], monthly income of less than 45.7 USD [AOR: 3.86, 95% CI: 1.62, 9.20], lack of hand washing after the use of the toilet with soap [AOR: 2.43, 95% CI: 1.22, 4.86] and untrimmed fingernails [AOR: 3.31, 95% CI: 1.99, 5.49] have significant association with intestinal parasitic infections.

**Conclusions:**

The high prevalence of intestinal parasitic infections in this study highlights the importance of food handlers as probable sources of parasitic infections. Public health measures and sanitation programs should be strengthened to control the spread of intestinal parasitic infections.

## Background

Intestinal parasitic infections (IPIs), caused either by intestinal protozoans or helminths or both, remain a major public health problem across the globe, particularly in low income countries due to difficulties in securing optimal hygienic food handling practices [1]. Centers for Disease Control and Prevention (CDC) estimates that each year, 48 million people get sick, 128,000 are hospitalized and 3000 dies from a foodborne disease [2]. Ethiopia has ranked the second highest burden of ascariasis, the third highest burden of hookworm and the fourth highest burden of trichuriasis in Sub-Saharan Africa [3].

The most prevalent intestinal protozoan parasites in Ethiopia are *Giardia lamblia* and *Entamoeba histolytica/ dispar*. Helminthic infection includes *Ascaris lumbricoides*, *Trichuris trichuria* and *Taenia saginata* [4, 5]. Many of these intestinal parasites usually cause asymptomatic infections or produce only mild symptoms, leading to difficulties in eradication and control [6].

Asymptomatic food handlers contribute significantly to the spread of infection to susceptible hosts, given that they are unaware of their potential to transmit, and therefore may not be practicing safe food handling [7]. Particularly, foods prepared in large quantities in high risk establishments such as University cafeterias are more liable to contamination when food handlers are shedding egg or cysts of parasite or food is cultivated in faeces-contaminated soils, fertilizer or water, and subsequently lead to outbreaks of food borne diseases [8]. The transmission could be effected from contaminated hand to the food prepared and finally, to healthy individuals through the chain of infection [2].

For the past two decades, Haramaya University (HU) has been experiencing rapid growth in student intake capacity and cafeteria facilities throughout its campuses. This dictates the need to ensure hygienic food handling and preparation practices in such cafeterias to safeguard the health and wellbeing of the customers. Moreover, the burden of IPIs and predisposing factors among food handlers is unknown. This study was designed to assess the prevalence of IPIs and associated factors among asymptomatic food handlers working at HU cafeterias.

## Methods

### Study design, period and area

A quantitative, cross-sectional study was conducted among asymptomatic food handlers at HU from August 2015 to January 2016. HU is located in East Hararghe zone at a distance of 510 kms from Addis Ababa, 17 kms from Harar town, and almost 5 kms off the main road from the nearby Haramaya town. The University comprises three campuses: Main, Harar and Chiro campus and consists of fifteen cafeterias (9 in Main, 4 in Harar and 2 in Chiro campus). During the study period, 1274 individuals were serving as food handlers throughout HU cafeterias.

### Study population

Study population consisted of asymptomatic food handlers working at HU cafeterias.

### Inclusion and exclusion criteria

All asymptomatic food handlers who had a direct contact with foods and drinks were included in this study. Those participants who took antiparasitic drugs in the last three weeks or during data collection were excluded.

### Sample size determination

The sample size was calculated using single population proportion formula considering 95% confidence level (CL), 5% margin of error, and a 52.4% prevalence of IPIs [9]. An initial sample size was 384. After considering 10% non-response, the final sample size was determined to be 422.

### Sampling techniques

**Fig. 1:**
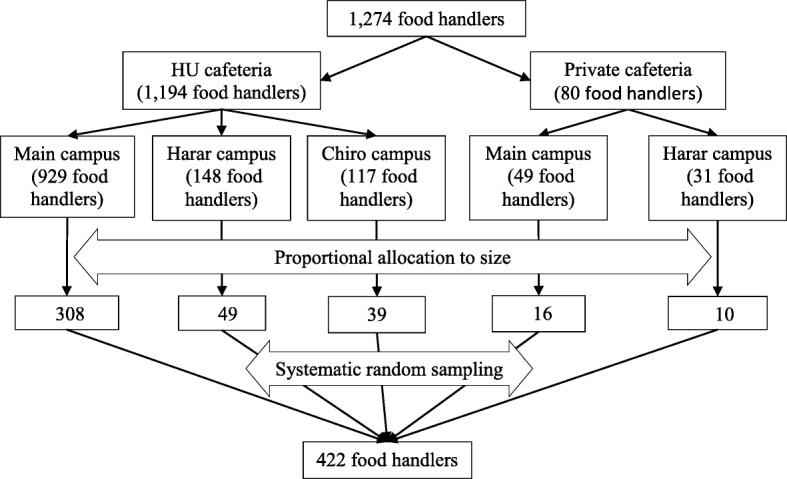
Flow chart showing sampling techniques

Food handlers were stratified into two strata based on the ownership of cafeteria (HU and private cafeteria). Food handlers working at HU cafeterias were further stratified by their working place (Main, Harar and Chiro campus). Food handlers from private cafeterias were also stratified similarly. Then, the sample size was proportionally allocated based on the size of food handlers working in each cafeteria. Finally, a sample size of 422 from a total population size of 1274 was selected using systematic random sampling technique (Fig. 1). A payroll of food handlers was used for sampling frame.

### Data and specimen collection

Data related to socio-economic factors, food handling practices and other related factors were collected using a pretested structured questionnaire administered by trained nurses. The questionnaire was adopted from the World Health Organization (WHO) food safety checklist and literatures [4, 10, 11]. Data on fingernail trimming was recorded by a simple observation. After interviewing and observation, respondents were asked to bring a small amount of fresh stool sample (2–3 g/pea-size if formed, or 4 ml if watery diarrhea) into a clean, tight-lid sample container after orienting on how to collect the stool specimen by attending laboratory technologist. All specimens were immediately transported using cold box to the Parasitology Laboratory of the College of Health and Medical Sciences of HU for analysis.

### Specimen processing and examination

Each stool sample was smeared and tested in triplicate. A saline and Lugol’s direct smears were performed by emulsifying small amount of stool (0.25 mg) uniformly in a drop of normal saline (0.85% NaCl) on one end of a glass slide, and Lugol’s iodine on the other end of the same slide. A saline direct stool smear was used for detection of motility of protozoan trophozoites, which were seen in semisolid/diarrheic specimens. Iodine direct smears showed the characteristic feature of the diagnostic stages in more details. Smear prepared from a sedimented stool was also examined to detect parasites that where too low to be seen in the direct wet smear and exclude false negative result in case of light infections, thus increasing the sensitivity of copromicroscopic techniques. Sedimentation technique was performed using Ridley modified formol ether sedimentation technique. In brief, 1 g of stool was placed in 15 ml conical tube containing 7 ml of 10% formol water, gently emulsified and sieved using 65 mm plastic strainer. The sieved sample was transferred into another conical tube containing 9 ml of 10% formol water and 3 ml of ethyl acetate and centrifuged at 3000 rpm. The supernatant was decanted by inverting the tube and the last drop allowed to sediment by gravity for 15 min. The sediment was then poured on slide, covered with cover glass (22 mm × 22 mm) and examined microscopically under low (10×) with the condenser iris closed sufficiently to give good contrast, and high (40×) objective lenses. Eggs and larvae of helminths, and cysts and trophozoites of protozoan were assessed by propagule size, shape, cell wall width and distinctive internal characteristics [12].

### Operational definition

*Asymptomatic infections* defined by the absence of acute gastrointestinal symptoms such as abdominal discomfort, vomiting, nausea and diarrhea [13].

*Food handler* is a person performing under contractual agreement or permanent employee who handles, prepares, serves, or sells food and drink, or who comes in contact with eating or cooking utensils or other equipment used in the handling, preparation, service, or sale of food.

### Data quality control

The questionnaire was initially prepared in English and translated into the local language (*Amharic* and *Afan Oromo*) by language experts and translated back into English by another expert and pretested on 5% asymptomatic food handlers working in Dire Dawa University, Eastern Ethiopia to ensure its consistency. The reliability of the questionnaire was validated using Cronbach’s alpha, and the result was 0.80, indicating a high level of internal consistency [14]. Data collectors and supervisors were trained for two days on method of data collection, specimen collection and examination techniques. The stool specimen examination was conducted in triplicate by trained Medical Parasitologists. The final result was determined through inter-examiners agreement. Moreover, investigators were involved in the decision in case of disagreement. Completion, accuracy and clarity of the collected data were checked every day.

### Methods of data analysis

Data were double entered into epidemiological information software (EPI Info™ version 3.5.1) to ensure accuracy of the data. The data were cleaned and exported to statistical package for social sciences (SPSS) software version 20.0 (SPSS Inc., Chicago, IL) for further analysis. Descriptive statistics were calculated for all variables. Bivariate and multivariable logistic regression models were used to assess the association between independent variables and IPIs. Variables that had a *p*-value < 0.25 in bivariate analysis were run in multivariable logistic regression at 95% CL to determine independent predictors of the outcome. A p-value < 0.05 was considered to indicate statistical significance association.

## Results

### Participant characteristics

This study included 417 asymptomatic food handlers (331 females and 86 males) with a response rate of 98.9%. The mean age of participants was 36.1 ± 8.7 standard deviation. Large proportion (39.3%) of food handlers were found in the age group of 31–40 years and had formal education (76.3%). Nearly half (47%) of the participants had more than 10 years of work experiences (Table 1).

**Table 1:** Characteristics of asymptomatic food handlers working at HU, eastern Ethiopia from August 2015 to January 2016 (*n* = 417)

Characteristics		Frequency	%
Sex	Female	331	79.4
	Male	86	20.6
Age (in year)	> 40	145	34.8
	31–40	165	39.3
	21–30	91	21.8
	< 21	17	4.1
Formal education	No	99	23.7
	Yes	318	76.3
Year of service (in year)	> 10	196	47.0
	5–10	93	22.3
	< 5	128	30.7
Average monthly income (in USD)	< 45.7	291	69.8
	45.7–91.3	27	6.5
	91.4–137	29	7.0
	> 137	70	16.8
Ownership of cafeteria	Private	77	18.5
	HU owned	340	81.5

### Prevalence of intestinal parasites

Of 417 stool specimens, 25.2% (95% CI: 18.3, 29.6) were found to be positive for one or more parasite species: comprising protozoa (61%) and helminths (39%). The most prevalent parasite was *E. histolytica/ dispar* (46.7%) followed by *A. lumbricoides* (14.3%) and then *G. lamblia* (13.3%). Less frequent identified intestinal parasite spp. were *T. trichuria, Schistosoma mansoni* and *Dientamoeba fragilis*; accounted for 2.9% of the total isolates (Table 2). Mixed intestinal parasites (*E. histolotica/dispar*, *G. lamblia* and *H. nana*) were detected in 2.6% of the participants.

**Table 2:** Percent of intestinal parasitic spp. isolated from the stools of the study participants at HU, eastern Ethiopia from August 2015 to January 2016 (*n* = 105)

Types of parasitic isolates	Frequency	%
*E. histolytica/ dispar*	49	46.7
*A. lumbricoides*	15	14.3
*G. lamblia*	14	13.3
*H. nana*	11	10.4
Hookworms	8	7.6
Taenia species	5	4.7
Others (*T. trichuria, S. mansoni, D. fragilis*)	3	2.9

### Factors associated with intestinal parasitic infections

Among the total variables included in the bivariate logistic analysis, ten variables (age, educational status, years of service, monthly income, hand washing after touching body parts, use of the apron when cooking, hand washing after the use of the toilet, fingernail trimming, hand washing before eating foods and medical check-up) were associated with IPIs at a *p*-value < 0.25. These variables were then computed in a multivariable logistic regression analysis. The results of multivariable logistic analysis showed that not attending formal education [AOR: 2.13, 95% CI: 1.24, 3.67], earning of a monthly income of less than 45.7 USD [AOR: 3.86, 95% CI: 1.62, 9.20], lack of hand washing after the use of the toilet with soap [AOR: 2.43, 95% CI: 1.22, 4.86] and untrimmed fingernails [AOR: 3.31, 95% CI: 1.99, 5.49] were significantly associated with IPIs (Table 3).

**Table 3:** Bivariate and multivariable logistic regression analysis on factors associated with IPIs among asymptomatic food handlers at HU, eastern Ethiopia from August 2015 to January 2016

Characteristics		Negative (%)	Posetive (%)	COR (95% CI)	AOR (95% CI)
Sex	Female	248 (74.9)	83 (25.1)	0.97 [0.57, 1.68] 1	–
	Male	64 (74.4)	22 (25.6)		
Age (in years)	> 40	98 (67.6)	47 (32.4)	2.11 [0.54, 7.00] 1.79 [0.97, 3.31] 1.05 [0.57, 1.93] 1	1.61 [0.44, 5.88] 1.77 [0.89, 3.51] 0.92 [0.48, 1.79] 1
	31–40	131 (79.9)	33 (20.1)		
	21–30	69 (75.8)	22 (24.2)		
	< 21	14 (82.2)	3 (17.6)		
Formal education	No	63 (63.6)	36 (36.4)	2.11 [1.29, 3.43] 1	2.13 [1.24, 3.67]* 1
	Yes	249 (78.3)	69 (21.7)		
Years of service (in years)	> 10	136 (69.4)	60 (30.6)	2.01 [1.17, 3.47] 1.42 [0.73, 2.73] 1	1.66 [0.91, 3.03] 1.57 [0.78, 3.20] 1
	5–10	71 (76.3)	22 (23.7)		
	< 5	105 (82.0)	23 (18.0)		
Average monthly income (in USD)	< 45.7	205 (70.4)	86 (29.6)	3.78 [1.66, 8.58] 3.15 [0.99, 10.07] 1.88 [0.54, 6.48] 1	3.86 [1.62, 9.20]* 3.22 [0.91, 11.43] 1.87 [0.50, 6.99] 1
	45.7–91.3	20 (74.1)	7 (25.9)		
	91.4–137	24 (82.8)	5 (17.2)		
	> 137	63 (90.0)	7 (10.0)		
Ownership of cafeteria	Private	61 (79.2)	16 (20.8)	0.74 [0.41, 1.35]	–
	HU	251 (73.8)	89 (26.2)		
Hand washing before food preparing with a soap	No	79 (71.2)	32 (28.8)	1.29 [0.79, 2.11] 1	–
	Yes	233 (76.1)	73 (23.9)		
Hand washing after touching body parts	No	150 (70.4)	63 (29.6)	1.62 [1.03, 2.54] 1	1.33 [0.81, 2.18] 1
	Yes	162 (79.4)	42 (20.6)		
Use of apron when handling food	No	109 (69.9)	47 (30.1)	1.51 [0.96, 2.37] 1	1.37 [0.83, 2.27] 1
	Yes	203 (77.8)	58 (22.2)		
Hand washing after toilet with a soap	No	231 (71.5)	92 (28.5)	2.48 [1.32, 4.68] 1	2.43 [1.22, 4.86] * 1
	Yes	81 (86.2)	13 (13.8)		
Fingernail trimming	No	81 (58.7)	57 (41.3)	3.39 [2.14, 5.36] 1	3.31 [1.99, 5.49] * 1
	Yes	231 (82.8)	48 (17.2)		
Hand washing before eating food with a soap	No	75 (72.8)	28 (27.2)	0.59 [0.69, 1.90] 1	–
	Yes	237 (75.5)	77 (24.5)		
Medical check-up (in the last 6 months)	No	223 (72.6)	84 (27.4)	1.59 [0.93, 2.73] 1	1.43 [0.79, 2.57] 1
	Yes	89 (80.9)	21 (19.1)		
Food safety training	No	219 (74.7)	74 (25.3)	1.10 [0.62, 1.64] 1	–
	Yes	93 (75.0)	31 (25.0)		

Note: *P < 0.05, *COR* Crude odds ratio, *AOR* Adjusted odds ratio, *CI* Confidence interval

## Discussion

The prevalence of IPIs in the present study was 25.2%. The finding was in agreement with a study report (25%) from Gonder University, Ethiopia [15], lower compared to previous studies conducted elsewhere in Ethiopia: Bahir Dar town (41.1%) [16], Yebu town (44.1%) [17], Addis Ababa (45.3%) [5] and Mekelle (52.4%) [9], but higher than a report (10.3%) in Sai-Yok, Thailand [18]. The existence of such variations may be explained by the differences in practices of personal hygiene, environmental sanitation, health promotion practices, geographical location and type of diagnostic sensitivity.

The most frequently isolated intestinal parasite in this study was *E. histolytica/ dispar* (46.7%). The finding was in line with two studies (32.3%) [9] and (36.6%) [10] conducted in different time period in Mekelle University, Ethiopia. But, it was in disagreement with a study report in Gonder University [15], in which *G. lamblia* (11%) was most predominantly isolated. The variations in the frequency and type of parasites might be due to differences in sample size (small sample size might overestimate the proportion), geographical location and environmental conditions.

The odds of being positive for atleast one IPIs was two times higher among food handlers who had no formal education than those who attended formal eduaction. This was supported by studies conducted elsewhere in Ethiopia [15, 19]. This may be explained in terms of lack of knowledge that made food handlers unaware of food safety guidelines and hence, may have reduced their understanding of the risks of parasitic contamination as well as protocols to mitigate these risks.

In this study, the odds of being infected with IPIs was four fold higher among food handlers who earned a monthly income of < 45.7 USD than those who earned > 134.0 USD. Similar findings were documented in other studies [19, 20]. The effect of low income on risk of parasitic infections is complex in nature and could be attributed to the sources of drinking water and food, environment sanitation, access to education and living conditions of individuals [21].

Food handlers who did not wash their hands after the use of the toilet had two time higher odds having IPIs compared to food handlers who did. This was supported by another study [10]. Not washing hands after the use of the toilet might have been affected by the availability of sanitary materials, level of education and lack of personal hygiene training, which highlight the need for future sanitation interventions. Conversely, lack of apron uses during food preparation, hand washing before food preparation, hand washing after touching body parts and lack of hand washing before eating food with a soap were not statistically associated with IPIs. These could be influenced by social desirability *bias.*

The odds IPIs was three fold higher among food handlars who had untrimmed fingernails compared to those who trimmed fingernails. Other studies have also shown untrimmed fingernails to be a determinant for IPIs among food handlers [17, 19]. Untrimmed fingernails could serve as a vehicle for transport of organisms from the source to the food due to the area beneath a fingernail harbors most organisms and is difficult to clean [22]. However, the present study did not attempt to assess the parasite carriage of the fingernail contents.

There were some limitations in this study. Firstly, the fingernail contents examination was not performed for ova/cyst of parasites. Examination of fingernail contents is one way to indicate cross-contamination and transferring of parasites from infected food handlers to actual food, and then to healthy individuals. Social desirability bias, which may cause weak association of hand washing habits with IPIs is another concern. In spite of these limitations, the use of sensitive diagnostic techniques and combination of methods with triplicate examination applied in this study would help to recover greater rate of intestinal parasites that would indicate the ‘true prevalence’.

## Conclusion

The prevalence of IPIs in this study is high. *E. histolytica/dispar* and *G. lamblia* are the most prevalent intestinal parasites. Not attending formal education, low monthly income, lack of hand washing after the use of the toilet with soap and untrimmed fingernails are independent predictors of IPIs. Preventive programs on awareness of the infectious diseases, improving hygiene and environmental sanitation should be strengthened to reduce IPIs. Large scale longitudinal study is recommended to robustly capture the burden of IPIs and its health effect on food handlers and customers.

## Abbreviations

AOR: Adjusted Odds Ratio
CDC: Centers for Disease Control and Prevention
CL: Confidence Level
COR: Crude Odds Ratio
HU: Haramaya University
IPIs: Intestinal Parasitic Infections
WHO: World Health Organization
